# Supplementary material for: Patterns of Intron Gain and Loss in Fungi
Source: PLoS Biol. 2004 Nov 30;2(12):e422. doi: 10.1371/journal.pbio.0020422 (PMC532390; doi:10.1371/journal.pbio.0020422)
Supplement: Table S1 — Also available at http://genes.mit.edu/NielsenEtAl/. (4.3 MB ZIP). [file pbio.0020422.st001.zip › NielsenEtAl/html/418.html]

AN0380.1.NCU09819.1.MG05731.1.FG00480.1


```
 CLUSTAL W (1.82) Multiple Sequence Alignments - Introns Inserted


Sequence 1: NCU09819.1	632 aa
Sequence 2: MG05731.1	667 aa
Sequence 3: FG00480.1	621 aa
Sequence 4: AN0380.1	629 aa
Alignment Length: 694 aa
Number Identitical Residues: 338 aa
Alignment Score (without introns) 16632


MG05731.1 	MCSRYMAVFGVYLAFKSTTAKRKMARSRKWGRSVRCDALPINTIANFWRSNAWNFILACL
NCU09819.1	--------------------------------------MESNKLENDVKS----------
FG00480.1 	--------------------------------------MASD---DDNRS----------
AN0380.1  	--------------------------------------MTAEEEQAKLAQ----------
          	                                      :  :       .          

MG05731.1 	DYNKQK1KMKSEGTDDDVKSLKRSHSETQDDD~D1NSSDDDMGPQLP-SQAPKKKRRVLP
NCU09819.1	-SKRTH~EEFTEGVAQDVS-----------GD~D1SSSDDDMGPQLPSAEAPKKKRRVLP
FG00480.1 	-QKRNH~TEFRDDAS-----------------~D1SSSDDDMGPQLP-SQAPKKKRRVLL
AN0380.1  	-NKRPH~SEVEADED----------------G1S~ESSDDDFGPALPSADAPKKKRRKLP
          	  :: :      .                  . . .*****:** **:::******* * 

MG05731.1 	YEKLYTSALPKSPRYSKSLMHKEQLAFVLWTPITEFLITVSIDGVVKFWKKIGEGIEFVK
NCU09819.1	YEKLYLKAMPKSARYSKSLMHKEQLTFLTMTPITDFLITTSIDGVVKFWKKVTGELEFVK
FG00480.1 	HEKLYVAALPKSPRYSKSLMHKEQILFATWTPLTEFLITSSIDGVVKFWKKIGQGIDFVK
AN0380.1  	FEKVYVNALPASARYSKSLMHKDQLSFVTMTPHTDFLITSSIDGVVKFWKKMAVGVEFVK
          	.**:*  *:* *.*********:*: *   ** *:**** ***********:   ::***

MG05731.1 	EFKAHAGEIKSVSVSADGRSFATAGLDKTVKIFDVITFD~LLAMVQLDYVPRCVCWVHKK
NCU09819.1	EYKAHLGEVKSVSVSQDGRSFATAGADKTIKLFDVNAFD~LLAVLQLDYVPACVCWVHRK
FG00480.1 	EFKAHDGEIKSVSVSNDGRSFATVGADETVKIFDVITFD~LLSMISLNYAPNCVCWVHSK
AN0380.1  	EFRAHAAEIKGVSVSADGRSFATTGADKTVKIFDVITFD1LLAMLTLDFSPRCICWVHPR
          	*::** .*:*.**** *******.* *:*:*:*** :** **::: *:: * *:**** :

MG05731.1 	GARLPLLAVSEFEKPLIHILDGRGEKTESIHTIKGLHRSPVTLMIFNDAYDCVISADQGG
NCU09819.1	GAPLPILAVSEEEKPVIHLYDGRGQQEEPFHTISGLHRSPVGIMAFNDRFDCVVSADDGG
FG00480.1 	GASVPVLAVSEESKPLIHIYDGRGEKEEAIHTIKGLHRKPVHLMAYNEAYDCVVSADEGG
AN0380.1  	GASLPLLAVTDDASSTIRIYDGRGENPAPLHTLKSVHRSPISAIAFNDAYNCVVSADESG
          	** :*:***::  .. *:: ****::  .:**:..:**.*:  : :*: ::**:***:.*

MG05731.1 	MLEYWRPNE-TYQKPGNVFAMKSSTNLFEFKKAKAVPTSLTISPNG--------------
NCU09819.1	MVEYWQPSG-SYGKPESVFRFKSATNLFEFKKAKAVPTSLALSPDGSRFVTVSFPDRKIR
FG00480.1 	MLEYWRPSG-DYEKPEGVFEYKSSTNLFDFKKAKSVPSCLSISPNGRSFVTFSLPDRKIR
AN0380.1  	MIEYWRASDGTFEKPDNVFELKSSTNLFEFKKAKSTPSSITISPSGKQFATISFPDRQVR
          	*:***:.. . : ** .**  **:****:*****:.*:.:::**.*    : : .. .  

MG05731.1 	---------ER2TYDESIRTVEEMQQAGTAIQKLDDNEFKRRLAQERELDAPALSDKINV
NCU09819.1	IFDFASGKLQR~TYDESLKVVEEMQQAGTALQKLDAVEFGRRIAQEREIESPALRNKFNV
FG00480.1 	MFDFATAKLHR~EYDESLQVAEEMHRAGTGATKLDNVEFGRRAAHERDIESDMLKYKTNV
AN0380.1  	VFDFGTGKLYR~KYDESLSTITDMQQAGTALYTLDAVEFGRRLAVERELENPVTKPKANV
          	  . .:..  *  ****: .  :*::***.  .**  ** ** * **:::      * **

MG05731.1 	IFDESGHFIMYGSILGVKVLNTYSNKVVKVYGKDENFRALNLAIYQGQPQKKGVTTVAMA
NCU09819.1	VFDESGHFILYGSYLGIKVLNTYTNQVAKVYGKEEGFRPLSLAIYQGQPQKKGVTTVAMA
FG00480.1 	IFDESGNFIIYGSMLGIKVLNTYTNQVVKVYGKDENFRAVNVAIYQGQPQKKGITTVEMG
AN0380.1  	IFDESNHFILYGSLYGVKCINTYTNRVVRVYAKDEPFRPLNLAMYQGQPQKKGVVTVSMA
          	:****.:**:***  *:* :***:*:*.:**.*:* **.:.:*:*********:.** *.

MG05731.1 	ASSNPLLQESEVRDPILVATGAGKGRFYMFTNDEDISKTTRDVQNEKP-TILGGAKKAAE
NCU09819.1	ASANPLLQESETRDPMLIATGVGKVRFYMFTNDEEISKSTRDVQNEKP-TVLGGPKKVEQ
FG00480.1 	ASVNPLLQEAETRDPMLVATGVGKVRFYMFTNEEEVSKSTRDVQNEKP-TMLGHKKDAKA
AN0380.1  	ASANPLLAEAEERDPILVTTGFAKLRFYLFTNETEISKSTRDVHNEKPRDIDSAAATAAS
          	** **** *:* ***:*::** .* ***:***: ::**:****:****  : .    .  

MG05731.1 	KK-AETGTSAIIHTSYGDIHIRMFPDAAPKAVENFVTHSKRGYYNNTIFHRVIRKFMIQC
NCU09819.1	KKAAETGTSAVIHTTYGDIHIRLFPDAAPKAVENFVTHAKRGYYNNTIFHRVIRKFMIQG
FG00480.1 	KK-TETGTAAILRTTYGDIHIRLFPDAAPKAVENFVTHSKNGYYNNTIFHRVIRKFMIQG
AN0380.1  	NKSGELGTSAILHTTMGDIHLRLFPSAAPKAVENFTTHAKNGYYNNTIFHRVIRKFMIQG
          	:*: * **:*:::*: ****:*:**.*********.**:*.****************** 

MG05731.1 	GDPLGDGTGGESIWGREFEDEFSSLKHDKPYTVSMANAGPNTNGSQFFITTEKT0SWLDG
NCU09819.1	GDPLGDGTGGESIWGKEFEDEFSSLKHDKPYTVSMANAGPNTNGSQFFITTEKT0PWLDN
FG00480.1 	GDPLGDGTGGESIWGREFEDEFSSLKHDKPFTVSMANAGPNTNASQFFITTEKT0PWLDG
AN0380.1  	GDPLGDGTGGESIWGGEFEDEFSALKHDKPYTLSMANAGPNTNGSQFFITTEKT~PWLDG
          	*************** *******:******:*:**********.********** .***.

MG05731.1 	KHTIFGRAVQGFDVIHKIENARTHKEKPEEDIKILNIDVF
NCU09819.1	KHTIFGRGVQGLDVIHRIENVKTHKDKPVEDIKILNIDIM
FG00480.1 	KHTIFGRATQGFDIIHKIENVRTHKEQPEEDIKIVNIDII
AN0380.1  	KHTIFGRAVQGLDVVHKIENVKTVKEKPEVDVKIVSISVS
          	*******..**:*::*:***.:* *::*  *:**:.*.:
```
